# Differential Changes in Akt and AMPK Phosphorylation Regulating mTOR Activity in the Placentas of Pregnancies Complicated by Fetal Growth Restriction and Gestational Diabetes Mellitus With Large-For-Gestational Age Infants

**DOI:** 10.3389/fmed.2021.788969

**Published:** 2021-12-06

**Authors:** Tai-Ho Hung, Chung-Pu Wu, Szu-Fu Chen

**Affiliations:** ^1^Department of Obstetrics and Gynecology, Taipei Chang Gung Memorial Hospital, Taipei, Taiwan; ^2^Department of Obstetrics and Gynecology, Keelung Chang Gung Memorial Hospital, Keelung, Taiwan; ^3^Department of Medicine, College of Medicine, Chang Gung University, Taoyuan, Taiwan; ^4^Graduate Institute of Biomedical Sciences, Department of Physiology and Pharmacology and Molecular Medicine Research Center, College of Medicine, Chang Gung University, Taoyuan, Taiwan; ^5^Department of Physical Medicine and Rehabilitation, Cheng Hsin General Hospital, Taipei, Taiwan

**Keywords:** placenta, fetal growth restriction, gestational diabetes mellitus, AMP-activated protein kinase, phosphoinositide 3-kinase, Akt

## Abstract

**Background:** Dysregulation of placental mechanistic target of rapamycin (mTOR) activity has been implicated in the pathophysiology of pregnancies complicated by idiopathic fetal growth restriction (FGR) and gestational diabetes mellitus (GDM) with large-for-gestational-age (LGA) infants. However, the underlying mechanisms remain unclear.

**Methods:** We obtained placentas from women with normal pregnancies (*n* = 11) and pregnancies complicated by FGR (*n* = 12) or GDM with LGA infants (*n* = 12) to compare the levels of total and phosphorylated forms of Akt, AMPK, TSC2, and mTOR among the three groups and used primary cytotrophoblast cells isolated from 30 normal term placentas to study the effects of oxygen–glucose deprivation (OGD) and increasing glucose concentrations on the changes of these factors *in vitro*.

**Results:** Placentas from FGR pregnancies had lower phosphorylated Akt (p-Akt) levels (*P* < 0.05), higher p-AMPKα levels (*P* < 0.01), and lower mTOR phosphorylation (*P* < 0.05) compared to that of normal pregnant women. Conversely, women with GDM and LGA infants had higher p-Akt (*P* < 0.001), lower p-AMPKα (*P* < 0.05), and higher p-mTOR levels (*P* < 0.05) in the placentas than normal pregnant women. Furthermore, primary cytotrophoblast cells subjected to OGD had lower p-Akt and p-mTOR (both *P* < 0.05) and higher p-AMPKα levels (*P* < 0.05) than those cultured under standard conditions, but increasing glucose concentrations had opposite effects on the respective levels. Administering compound C, an AMPK inhibitor, did not significantly affect Akt phosphorylation but partially reversed mTOR phosphorylation. Administering LY294002, an Akt inhibitor, decreased p-mTOR levels, but did not change the levels of total and phosphorylated AMPKα.

**Conclusion:** These results suggest that Akt and AMPK are involved in the regulation of trophoblast mTOR activity in the placentas of pregnancies complicated by FGR and GDM with LGA infants.

## Introduction

Idiopathic fetal growth restriction (FGR) and gestational diabetes mellitus (GDM) are common pregnancy complications (1, 2). Growth-restricted fetuses are at increased risk of perinatal morbidity and mortality (1). On the other hand, fetuses of GDM pregnancies are more likely to be large-for-gestational age (LGA) and encounter birth injuries and neonatal complications such as hypoglycemia, hyperbilirubinemia, and respiratory distress (3). Newborns of both pregnancy complications are also at increased risk for metabolic disorders, including obesity, type 2 diabetes mellitus, and cardiovascular diseases later in life (3–5).

Although the etiologies of FGR and GDM are not fully understood, abnormal placental development and functional dysregulation may play essential roles in mediating suboptimal or overgrowth of the fetuses in these pregnancy complications (6–8). Compared with placentas from women with normal pregnancies, placentas from FGR pregnancies are usually smaller and lighter, whereas placentas from GDM pregnancies, particularly those with LGA infants, are usually bigger and heavier, suggesting that there are differences in trophoblast cell proliferation, differentiation, and death in these two complications (6, 9). Indeed, differential changes in trophoblast proliferation, apoptosis, and autophagy in the placenta have been noted between women with normal pregnancies and those with pregnancies complicated by FGR or GDM with LGA infants (6, 10–15).

A key component regulating the balance between cell growth, apoptosis, and autophagy in response to cellular physiological conditions and environmental stress is the mechanistic target of rapamycin (mTOR) (16–18). In human placentas, mTOR is localized mainly in the trophoblast layer and can exist as one of two complexes, mTORC1 and mTORC2, that are regulated by distinct mechanisms and have distinct functions (17, 19). Both amino acid transporter levels and mTORC1 activity are reduced in the placentas of FGR pregnancies (12, 18–21), while increased mTORC1 activity, indicated by increased levels of its target proteins, such as p70S6K and 4E-BP-1, was noted in the placentas from GDM pregnancies with LGA infants (22, 23), compared to that in the placentas of normal pregnancies. Nevertheless, the mechanistic models of signaling pathways involved in regulating mTORC1 activity in the placentas of these two pregnancy complications remain unclear.

Protein kinase B, also known as Akt, and AMP-activated protein kinase (AMPK) are two molecules suggested as important sensors of changes of growth factors, nutrient signals, and metabolic stress in cells (24). Upon activation by phosphoinositide 3-kinase (PI3K) in response to growth factor signaling, Akt further activates mTOR by reducing the inhibitory effects of the tuberous sclerosis complex (TSC1/TSC2) on mTORC1 (25). AMPK is a heterotrimer complex, including a catalytic subunit (α) and two regulatory subunits (β and γ). During energy depletion, AMPK is activated and it directly phosphorylates TSC2 or the critical mTORC1 binding subunit Raptor, thus inhibiting mTORC1 activity (26). Thus, we hypothesized that there are differential changes in the activities of Akt and AMPK, and would like to determine their phosphorylation levels in the placentas, between normal pregnancies and pregnancies complicated by FGR and GDM with LGA infants. Here, our objectives were to compare the levels of total and phosphorylated forms of Akt, AMPK, TSC2, and mTOR in the placentas of women with normal pregnancies and infants with weights appropriate for gestational age (AGA) and those of women with pregnancies complicated by FGR or GDM with LGA infants. Additionally, we investigated the effects of oxygen–glucose deprivation (OGD) and increasing glucose concentrations on the levels of total and phosphorylated forms of Akt, AMPK, TSC2, and mTOR in cytotrophoblast cells obtained from normal term placentas. By analyzing placental samples from pregnant women with different phenotypes of fetal growth and using primary cytotrophoblast to test the effects of OGD and increasing glucose concentrations on the changes of phosphorylation of Akt, AMPK, TSC2, and mTOR *in vitro*, we demonstrated that Akt and AMPK are important regulators for trophoblast mTOR activity in the placentas of pregnancies complicated by FGR and GDM with LGA infants.

## Materials and Methods

This study was approved by the Institutional Review Board of Chang Gung Memorial Hospital, Taiwan (201702210B0). All placental samples were collected from participants who provided written informed consent.

### Collection of Placental Tissues

We obtained placentas from singleton women with normal term pregnancies (*n* = 11) with AGA infants and pregnancies complicated by idiopathic FGR (*n* = 12) or GDM with LGA infants (*n* = 12). All these women had cesarean deliveries with indications including prior cesarean section or myomectomy and fetal malpresentation (for normal pregnancies), induction failure and abnormal intrapartum fetal heart rate patterns (for FGR pregnancies), and cephalopelvic disproportion and macrosomia (for GDM with LGA infants). FGR is defined as a birth weight below the 10th percentile, while LGA is defined as a birth weight above the 90th percentile of the mean weight corrected for fetal sex and gestational age, respectively. Infants with a birth weight between the 10th and 90th percentiles of the mean weight corrected for fetal sex and gestational age were considered AGA. The diagnosis of idiopathic FGR was attributed to one probable placental cause after excluding other major identifiable causes such as fetal chromosomal or structural abnormalities, maternal malnutrition, infection, and substance abuse. Based on the results of a 75 g, 2 h oral glucose tolerance test, GDM was diagnosed when one or more of the following plasma glucose levels were met or exceeded: fasting, 92 mg/dl; 1 h, 180 mg/dl; or 2 h, 153 mg/dl. These women did not have any other medical diseases, such as chronic or gestational hypertension, preeclampsia, renal, thyroid, or autoimmune diseases.

Villous tissue samples were randomly collected from five distinct sites of the basal plate after delivery. Each site was midway between the cord insertion site and the placenta periphery, and midway between the chorionic and basal plates. The villous samples were immediately rinsed in ice-cold phosphate-buffered saline to clear the maternal blood and thereafter snap frozen in liquid nitrogen before being stored at −70°C for further processing. All villous samples were collected and processed within 10 min of delivery.

### Isolation and Culture of Cytotrophoblast Cells From Normal Term Placentas

We isolated cytotrophoblast cells from 30 normal term placentas, as previously described (12, 13). Briefly, the purified cells were plated in 6-well plates at a minimum density of 4 × 10^6^ cells/cm^2^ and cultured in Dulbecco's Modified Eagle Medium (DMEM; Gibco; catalog no. 12320032; Thermo Fisher Scientific, Inc., Waltham, MA, USA) containing 5.6 mM D-glucose, 5% fetal bovine serum, 100 U/ml penicillin, 100 μg/ml streptomycin, and 0.25 μg/ml amphotericin B (antibiotic-antimycotic, catalog no. 15240062; Gibco) in a humidified atmosphere with 5% CO_2_ and balanced air. After incubating overnight, the cells were rinsed twice with pre-warmed medium to remove non-attached cells and thereafter used in individual experiments.

### Oxygen–Glucose Deprivation (OGD) and Hyperglycemic Conditions

To study the effects of reduced oxygen and glucose concentrations on the levels of total and phosphorylated forms of Akt, AMPK, TSC2, and mTOR, primary cytotrophoblasts were cultured in RPMI 1,640 media in two conditions: with 5.6 mM D-glucose and 5% CO_2_/balanced air (defined as the standard conditions) and without glucose (catalog no. 11879; Invitrogen) in 2% O_2_ with 5% CO_2_/balanced N_2_ (defined as the OGD conditions), as previously described (12). After 24 h of incubation, the cells were collected, homogenized, and stored at −70°C for further processing.

To study the effects of increasing glucose concentrations on the changes of total and phosphorylated forms of Akt, AMPK, TSC2, and mTOR, primary cytotrophoblasts were cultured in a humidified chamber with 5% CO_2_/balanced air and DMEM medium with one of the following three glucose concentrations: (1) 5.6 mM D-glucose (defined as the standard conditions), (2) 5.6 mM D-glucose and 19.4 mM L-glucose (defined as the hyperosmotic conditions), and (3) 25 mM D-glucose (defined as the hyperglycemic conditions), as previously described (13). After 24 h of incubation, the cells were collected, homogenized, and stored at −70°C for further processing.

### Effects of Inhibition of AMPK and Akt on the Changes in mTOR Phosphorylation Levels

It has been proposed that there is crosstalk between Akt and AMPK in cells when facing metabolic stress (24). Therefore, specific inhibitors of AMPK (compound C, 10 μM in DMSO; EMD Millipore, Billerica, MA, USA) and Akt (LY294002, 10 μM in DMSO; Cell Signaling Technology, Danvers, MA, USA) were administered to primary cytotrophoblast cells cultured under OGD and hyperglycemic conditions for 24 h, respectively. After individual experiments, the cells were collected, homogenized, and stored at −70 °C for further processing to evaluate the changes in mTOR phosphorylation levels. The working concentrations of compound C and LY294002 were determined according to previous reports and preliminary experiments (27, 28).

### Western Blot Analysis

Western blotting was performed as described previously (12). Briefly, 50–100 μg of cytosolic protein sample per lane was separated by 12 or 16% sodium dodecyl sulfate polyacrylamide gel electrophoresis, transferred to nitrocellulose membranes, and probed with primary antibodies overnight at 4°C. Horseradish peroxidase-linked donkey anti-rabbit or sheep anti-mouse secondary antibodies were used in conjugation with enhanced chemiluminescence (SuperSignal West Pico, Pierce Chemical Company, Rockford, IL, USA) to visualize the bands of target proteins on autoradiography films. The relative intensities of the protein signals were normalized to the intensities of β-actin (clone AC-15, 1:10000 dilution; Sigma) signals, and the band densities were quantified by densitometric analysis using ImageJ software (National Institutes of Health, Bethesda, MD, USA; http://rsb.info.nih.gov/ij/). The source and working concentrations of each primary antibody used are listed in Table 1.

**Table 1:** Primary antibodies used in the western blots.

**Antibody**	**Source**	**Type**	**Catalog no**.	**Dilution**
Akt	Rabbit	Polyclonal	9272	1:2000
Phospho-Akt (Ser473)	Rabbit	Polyclonal	9271	1:500
AMPKα	Rabbit	Polyclonal	2532	1:2000 (tissue homogenates); 1:1000 (cell lysate)
Phospho-AMPKα (Thr172) (40H9)	Rabbit	Monoclonal, IgG	2535	1:1000
Tuberin/TSC2 (28A7)	Rabbit	Monoclonal, IgG	3635	1:2000
Phospho-Tuberin/TSC2 (Thr1462) (5B12)	Rabbit	Monoclonal, IgG	3617	1:1000
Phospho-Tuberin/TSC2 (Thr1571)	Rabbit	Polyclonal	3614	1:500 (tissue homogenates); 1:1000 (cell lysate)
mTOR	Rabbit	Polyclonal	2972	1:2000
Phospho-mTOR (Ser2448)	Rabbit	Polyclonal	2971	1:1000
Phospho-mTOR (Ser2481)	Rabbit	Polyclonal	2974	1:1000
p70S6 kinase	Rabbit	Polyclonal	9202	1:200
Phospho-p70S6 kinase (Thr389)	Rabbit	Polyclonal	9205	1:200
4E-BP1	Rabbit	Polyclonal	9452	1:2000
Phospho-4E-BP1 (Thr37/46)	Rabbit	Polyclonal	9459	1:2000
Phospho-4E-BP1 (Thr70)	Rabbit	Polyclonal	9455	1:1000
Raptor (24C12)	Rabbit	Monoclonal, IgG	2280	1:1000
Phospho-Raptor (Ser792)	Rabbit	Polyclonal	2083	1:1000

*All antibodies were obtained from Cell Signaling Technology, Danvers, MA, USA*.

The following method was adopted to compare the levels of phosphorylation of target proteins between samples: (1) One of the samples in women with normal pregnancies or in the control group of the culture experiments was assigned as the “standard of comparison (SC)”. Samples of SC were included in each run of gel electrophoresis and subsequent blotting; (2) Each sample's target protein optical density (OD) (including SC) was divided by its β-actin to normalize loading variation. Taking Akt as an example, this gave a value of S1_Akt_/S1_β−*actin*_ (for Sample no. 1) or SC_Akt_/SC_β−*actin*_ (for SC); (3) Similarly, OD of the phosphorylated form of the target protein for every sample (including SC) was divided by its OD of β-actin to normalize loading variation. Taking p-Akt as an example, this gave a value of S1_p−Akt_/S1_β−*actin*_ (for Sample no. 1) or SC_p−Akt_/SC_β−*actin*_ (for SC); and (4) The value in Step 3 was divided by the value in Step 2 to compare the change of target protein phosphorylation between the samples. For example, S1_p−Akt_/S1_β−*actin*:_ S1_Akt_/S1_β−*actin*_ was calculated first, and then divided by the ratio of SC_p−Akt_/SC_β−*actin*:_ SC_Akt_/SC_β−*actin*_. This calculated the relative change in the amount of phosphorylation of target protein for individual sample in comparison to the result of SC. Since samples of SC were included in each run of gel electrophoresis and blotting, differences between different samples were computed based on their relative changes in comparison to the levels of SC.

### Statistical Analysis

All data were analyzed and plotted using Prism 7 for Mac OS X (GraphPad Software, Inc., La Jolla, CA, USA). The clinical data of women enrolled in this study are presented as mean ± standard deviation, number (percentage), or median (range) when appropriate. The data from cell culture experiments are presented as mean ± SEM. All data were first tested for the homogeneity of variance (Bartlett's test) and normality (Kolmogorov-Smirnov test). The differences between two groups were determined using the Student's *t*-test or χ^2^ test. The differences between three groups were computed with one-way analysis of variance or non-parametric tests (Kruskal-Wallis test), followed by Tukey's *post-hoc* test if significant effects were determined. Results with *P* < 0.05 were considered statistically significant.

## Results

### Characteristics of the Study Population

The characteristics of the study population are presented in Table 2. There were no significant differences in the mean maternal age, pre-pregnancy body mass index, blood pressure before delivery, hemoglobin level, platelet count, and gestational age between the three groups. The rates of primiparity and male fetuses, and one-min and five-min Apgar scores were also similar. Nevertheless, women with GDM and LGA infants had higher mean 1 h and 2 h plasma glucose concentrations at 75 g oral glucose tolerance tests than women with normal or FGR pregnancies. Women with GDM and LGA infants also had higher mean birth weights and placental weights than the other two groups. In contrast, women with pregnancies complicated by FGR had lower mean birth weights and placental weights than women with normal pregnancies or women with GDM and LGA infants. Two women with pregnancies complicated by FGR were associated with abnormal Doppler velocimetry of the umbilical artery blood flow (absence of end-diastolic flow velocity). Four women with GDM received insulin treatment in addition to nutritional therapy.

**Table 2:** Characteristics of the study population.

	**Normal pregnancy**	**FGR**	**GDM with LGA infants**	***P***
	**(*n =* 11)**	**(*n =* 12)**	**(*n =* 12)**	
Age (y)	35.1 ± 4.5	33.3 ± 3.4	35.0 ± 3.5	0.47
Primiparity	5 (46%)	9 (75%)	8 (67%)	0.32
Pre-pregnancy body mass index (kg/m^2^)	20.7 ± 1.7	20.1 ± 1.9	22.8 ± 4.0	0.06
**Blood pressure before delivery**				
Systolic (mm Hg)	115.5 ± 13.0	123.8 ± 6.0	120.4 ± 10.8	0.17
Diastolic (mm Hg)	73.5 ± 8.7	74.5 ± 8.4	74.1 ± 8.9	0.96
Hemoglobin (g/dl)	11.2 ± 1.5	11.7 ± 1.3	11.5 ± 1.0	0.63
Platelet count (10^3^/μl)	217.1 ± 59.6	236.2 ± 60.0	222.0 ± 60.6	0.73
**Glucose level at 75-g OGTT (mg/dl)**				
Fasting	82.4 ± 4.6	81.6 ± 5.8	84.9 ± 6.5	0.35
1-h	135.4 ± 24.6	123.7 ± 27.2	182.1 ± 28.0^a^, ^c^	<0.01
2-h	118.5 ± 17.7	112.9 ± 19.0	163.4 ± 14.4^b^, ^c^	<0.01
Gestational age (wk)	38.6 ± 0.6	38.6 ± 0.8	39.4 ± 1.1	0.07
Birth weight (g)	3191.4 ± 134.1	2235.7 ± 217.4^b^	3932.1 ± 208.3^b^, ^c^	<0.01
Birth weight percentile	52.9 ± 11.9	4.6 ± 0.8^b^	99.1 ± 9.0^b^, ^c^	<0.01
Placental weight (g)	617.3 ± 78.8	441.8 ± 61.7^b^	818.8 ± 97.2^b^, ^c^	<0.01
One-minute Apgar score	9 (9–10)	9 (8–9)	9 (9–10)	0.33
Five-minute Apgar score	10 (9–10)	10 (9–10)	10 (9–10)	0.99
Male fetus	6 (55%)	5 (50%)	7 (58%)	0.92

*OGTT, oral glucose tolerance test; FGR, fetal growth restriction; GDM, gestational diabetes mellitus; LGA, large-for-gestational age. Data presented as the mean ± standard deviation, n (%), or median (range). P values based on χ^2^ test, one-way analysis of analysis of variance, or Kruskal-Wallis test.*

a *P < 0.01;*

b *P < 0.001, compared to women with normal pregnancies by Tukey's post-hoc test.*

c *P < 0.001, compared to women with FGR pregnancies by Tukey's post-hoc test*.

### Differences in Phosphorylated Akt and AMPKα Levels in Placentas

**Figure 1:**
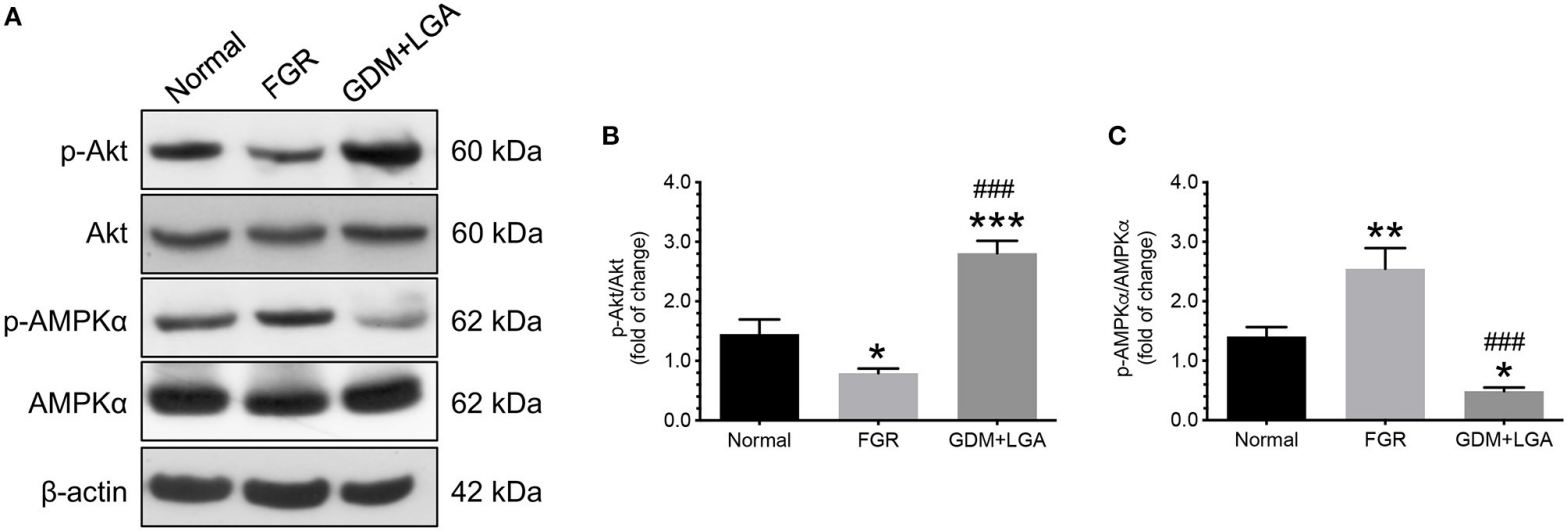
Differential changes in the levels of phosphorylated and total forms of Akt and AMPKα in villous samples obtained from women with normal pregnancies and women with pregnancies complicated by FGR or GDM with LGA infants. Placentas from women with pregnancies complicated by FGR had a significantly lower phosphorylated to total Akt ratio but a higher phosphorylated to total AMPKα ratio compared to the placentas from normal pregnant women. In contrast, GDM women with LGA infants had a higher placental phosphorylated to total Akt ratio, but a lower phosphorylated to total AMPKα ratio than those of the women with normal pregnancies or pregnancies complicated by FGR. **(A)** Representative blots show the levels of phosphorylated and total forms of Akt and AMPKα. β-actin was used to normalize loading variability. **(B,C)** Data are presented as mean ± SEM and *P* values are based on one-way ANOVA followed by Tukey's *post-hoc* test. *, *P* < 0.05; **, *P* < 0.01; and ***, *P* < 0.001, compared with normal pregnant women; ^###^, *P* < 0.001 compared to women with pregnancies complicated by FGR. Normal, normal pregnant women (*n* = 11); FGR, women with pregnancies complicated by FGR (*n* = 12); GDM+LGA, GDM women with LGA infants (*n* = 12).

Firstly, we measured the levels of phosphorylated and total forms of Akt and AMPKα in villous samples obtained from women with normal pregnancies and those from women with pregnancies complicated by FGR or GDM with LGA infants. The ratios of phosphorylated to total forms of Akt and AMPKα were computed and compared among the three groups of women. As shown in Figure 1, placental samples from women with pregnancies complicated by FGR had a significantly lower phosphorylated to total Akt ratio but a higher phosphorylated to total AMPKα ratio compared to that of normal pregnant women (Figure 1B). In contrast, GDM women with LGA infants had a higher placental phosphorylated to total Akt ratio, but a lower phosphorylated to total AMPKα ratio than those of the women with normal pregnancies or pregnancies complicated by FGR (Figure 1C).

### Differences in Phosphorylation Levels of TSC2 and mTOR in Placentas

**Figure 2:**
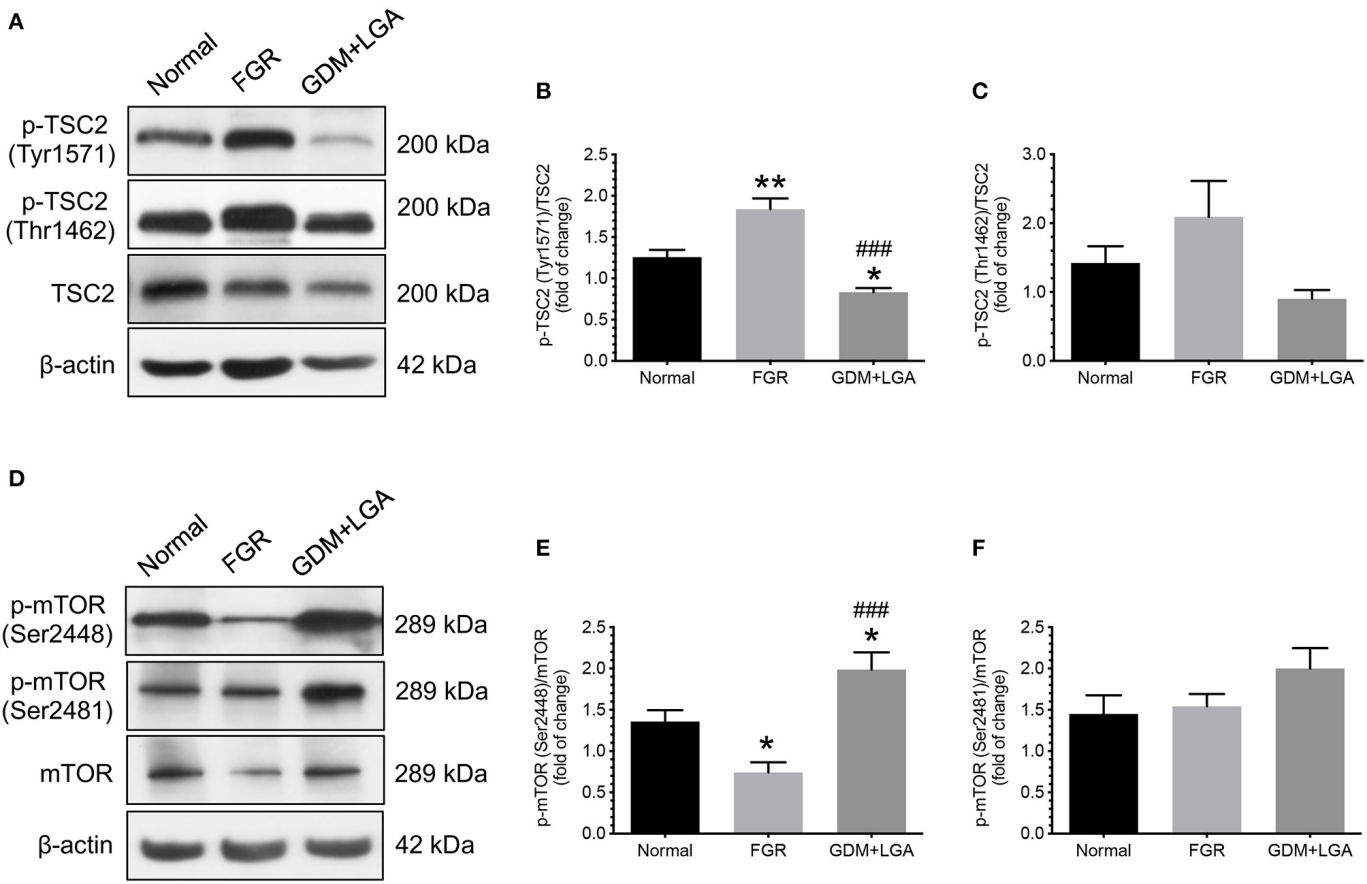
Differential changes in the phosphorylation of TSC2 and mTOR between placentas from normal pregnant women and those from pregnancies complicated by FGR or GDM with LGA infants. An increased phosphorylation of TCS2 (Tyr1571) was noted in the placentas of women with pregnancies complicated by FGR compared to that of normal pregnant women **(A,B)**. This change was associated with a decrease in phosphorylated mTOR levels (Ser2448) **(D,E)**. In contrast, GDM women with LGA infants had lower levels of TSC2 (Tyr1571) but higher levels of mTOR (Ser2448) in their placentas compared to that of normal pregnant women and women with FGR pregnancies **(A,B,D,E)**. There were no significant differences in the levels of phosphorylated forms of TSC2 (Thr1462) and mTOR (Ser2481) among the three groups of women **(A,C,D,F)**. **(A,D)** Representative blots show the levels of phosphorylated and total forms of TSC2 and mTOR. β-actin was used to normalize loading variability. **(B,C,E,F)** Data are presented as mean ± SEM and *P* values are based on one-way ANOVA followed by Tukey's *post-hoc* test. *, *P* < 0.05; **, *P* < 0.01, compared with normal pregnant women; ^###^, *P* < 0.001 compared to women with pregnancies complicated by FGR. Normal, normal pregnant women (*n* = 11); FGR, women with pregnancies complicated by FGR (*n* = 12); GDM+LGA, GDM women with LGA infants (*n* = 12).

We also studied the changes in downstream effectors of the Akt and AMPK signaling pathways. TSC2-related and mTOR-related molecules were chosen as they have been suggested to play essential roles in placental development and fetal growth (18, 29). As shown in Figure 2, an increased phosphorylation of TCS2 (Tyr1571) was noted in the placentas of women with pregnancies complicated by FGR compared to that of normal pregnant women (Figures 2A,B). This change was associated with a decrease in phosphorylated mTOR levels (Ser2448) (Figures 2D,E). In contrast, GDM women with LGA infants had lower levels of TSC2 (Tyr1571) but higher levels of mTOR (Ser2448) in their placentas compared to that of normal pregnant women and women with FGR pregnancies (Figures 2A,B,D,E). There were no significant differences in the levels of phosphorylated forms of TSC2 (Thr1462) and mTOR (Ser2481) among the three groups of women.

### Differences in Phosphorylation Levels of Akt, AMPKα, TSC2, and mTOR in Cells Treated With OGD and at Standard Conditions

**Figure 3:**
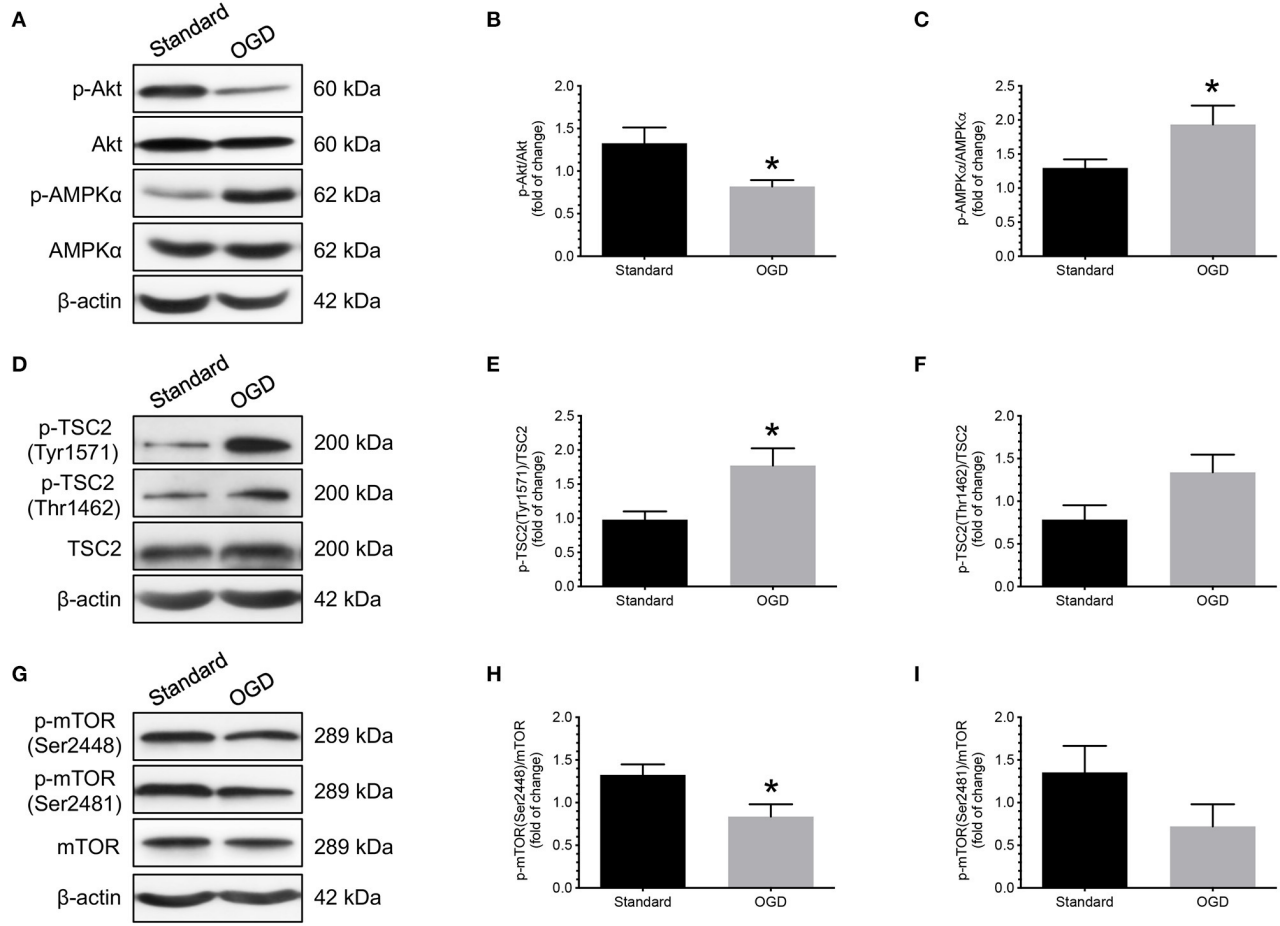
Effects of OGD on the changes of Akt, AMPKα, TSC2, and mTOR phosphorylation in human cytotrophoblast cells. Cytotrophoblast cells were isolated from normal term placentas and cultured at either OGD or standard conditions for 24 h. Cytotrophoblast cells subjected to OGD treatment had decreased phosphorylation of Akt and increased phosphorylation of AMPKα than the cells cultured under standard conditions **(A–C)**. OGD treatment also led to increased TSC2 (Tyr1571) phosphorylation **(D,E)** and decreased mTOR (Ser2448) phosphorylation **(G,H)**. There were no significant differences in the measurements of p-TSC2 (Thr1462)/TSC2 and p-mTOR (Ser2481)/mTOR between these two groups **(F,I)**. **(A,D,G)** Representative blots show the levels of phosphorylated and total forms of Akt, AMPKα, TSC2, and mTOR. β-actin was used to normalize loading variability. **(B,C,E,F,H,I)** Data are presented as mean ± SEM; *n* = 8 for each group. *, *P* < 0.05; compared with cells cultured at standard conditions based on Student's *t*-test. Standard, standard culture conditions; OGD, oxygen-glucose deprivation.

Next, we used an *in vitro* model that we previously established to study the effects of OGD on the phosphorylation of Akt, AMPKα, TSC2, and mTOR in human cytotrophoblast cells (12). Primary cytotrophoblast cells were isolated from normal term placentas and cultured under either OGD or standard conditions for 24 h. Changes in Akt, AMPKα, TSC2, mTOR, and their associated phosphorylated forms were evaluated by western blotting. As shown in Figure 3, cytotrophoblast cells subjected to OGD treatment had decreased phosphorylation of Akt and increased phosphorylation of AMPKα than the cells cultured under standard conditions (Figures 3A–C). These changes were associated with increased TSC2 (Tyr1571) phosphorylation (Figures 3D,E) and decreased mTOR (Ser2448) phosphorylation (Figures 3G,H). There were no significant differences in the measurements of p-TSC2 (Thr1462)/TSC2 and p-mTOR (Ser2481)/mTOR between these two groups (Figures 3D,F,G,I).

### Differences in Phosphorylation Levels of Akt, AMPKα TSC2, and mTOR in Cells Treated With Normoglycemic, Hyperosmotic, and Hyperglycemic Conditions

**Figure 4:**
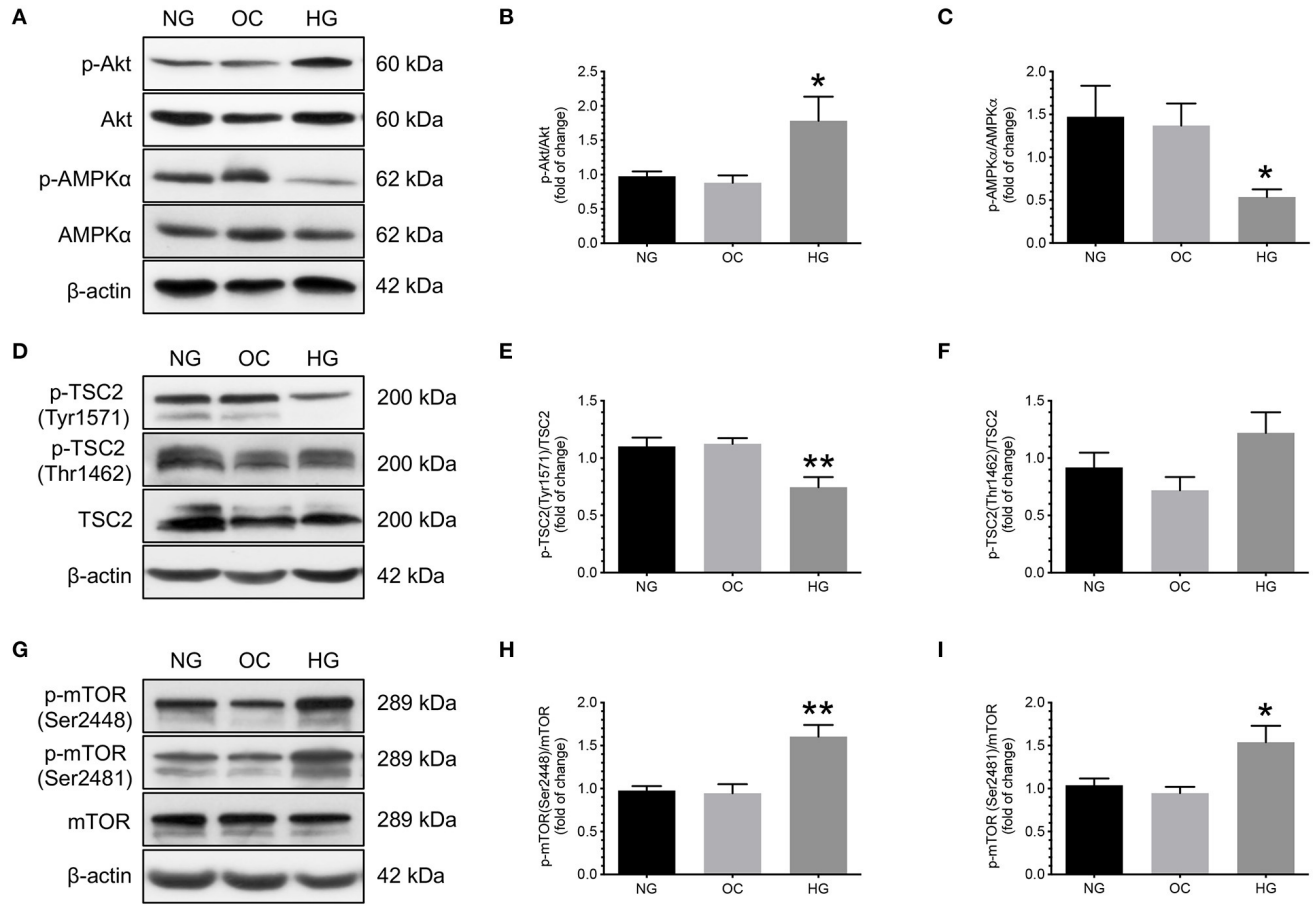
Effects of increasing glucose concentration on the changes of Akt, AMPKα, TSC2, and mTOR phosphorylation in human cytotrophoblast cells. Primary cytotrophoblast cells were cultured under normoglycemic, hyperosmotic, and hyperglycemic conditions for 24 h, and the levels of phosphorylated and total forms of Akt, AMPKα, TSC2, and mTOR were compared. Increasing D-glucose concentration led to higher p-Akt/Akt and lower p-AMPK/AMPKα than that of those under normoglycemic and hyperosmotic conditions **(A–C)**. These changes were concomitant with a decrease in p-TSC2 (Tyr1571) levels and an increase in mTOR phosphorylation (Ser2448 and Ser2481) **(D,E,G–I)**. No significant differences in the phosphorylated levels of TSC2 (Thr1462) were noted among the three experimental groups **(D,F)**. **(A,D,G)** Representative blots show the levels of phosphorylated and total forms of Akt, AMPKα, TSC2, and mTOR. β-actin was used to normalize loading variability. **(B,C,E,F,H,I)** Data are presented as mean ± SEM; *n* = 9 for each group. *, *P* < 0.05; **, *P* < 0.01; compared with cells cultured at normoglycemic conditions based on one-way analysis of variance followed by Tukey's *post-hoc* test. NG, normoglycemic conditions; OC, hyperosmotic conditions; and HG, hyperglycemic conditions.

To investigate the effect of increasing glucose concentrations on the changes in phosphorylation of Akt, AMPKα, TSC2, and mTORC1, primary cytotrophoblast cells were cultured under normoglycemic, hyperosmotic, and hyperglycemic conditions for 24 h, and the levels of phosphorylated and total forms of Akt, AMPKα, TSC2, and mTOR were compared. Increasing D-glucose concentration led to higher p-Akt/Akt and lower p-AMPK/AMPKα than that of those under normoglycemic and hyperosmotic conditions (Figures 4A–C). These changes were concomitant with a decrease in p-TSC2 (Tyr1571) levels and an increase in mTOR (Ser2448 and Ser2481) phosphorylation (Figures 4D,E,G–I). No significant differences in the phosphorylated levels of TSC2 (Thr1462) were noted among the three experimental groups (Figure 4F).

To further confirm the specificity of mTORC1 activation, we compared the levels of phosphorylated and total forms of mTORC1 downstream proteins 4E-BP1 and p70S6K. As shown in the Supplementary Figure 1, increasing glucose concentrations led to higher levels of phosphorylation of 4E-BP1 at Thr-37/46 and Thr-70 and the ratios of p-4E-BP1 (Thr37/46)/4E-BP1 and p-4E-BP1 (Thr70)/4E-BP1 (Supplementary Figures 1a–c). Hyperglycemia also caused a higher p-p70S6K (Thr389)/p70S6K than the normoglycemic and hyperosmotic conditions (Supplementary Figures 1a,d).

### Effects of OGD and Increasing Glucose Concentrations on the Phosphorylation of Raptor in Primary Cytotrophoblast Cells

In addition to regulating mTOR via TSC2 phosphorylation, AMPK has also been reported to modulate mTORC1 activity by phosphorylating Raptor at Ser-792. To explore the possibility of this regulating mechanism, cytotrophoblast cells were cultured under standard and OGD conditions, or normoglycemic, hyperosmotic, and hyperglycemic conditions for 24 h, respectively, and the levels of phosphorylated and total forms of Raptor were compared. As demonstrated in the Supplementary Figure 2a, OGD caused a higher p-Raptor (Ser792)/Raptor ratio compared with the standard culture conditions. In contrast, increasing D-glucose concentration led to lower levels of p-Raptor (Ser792) than that of those under normoglycemic and hyperosmotic conditions (Supplementary Figure 2b).

### Effects of Compound C on Phosphorylation Levels of Akt and mTOR in Cells Under OGD Conditions

**Figure 5:**
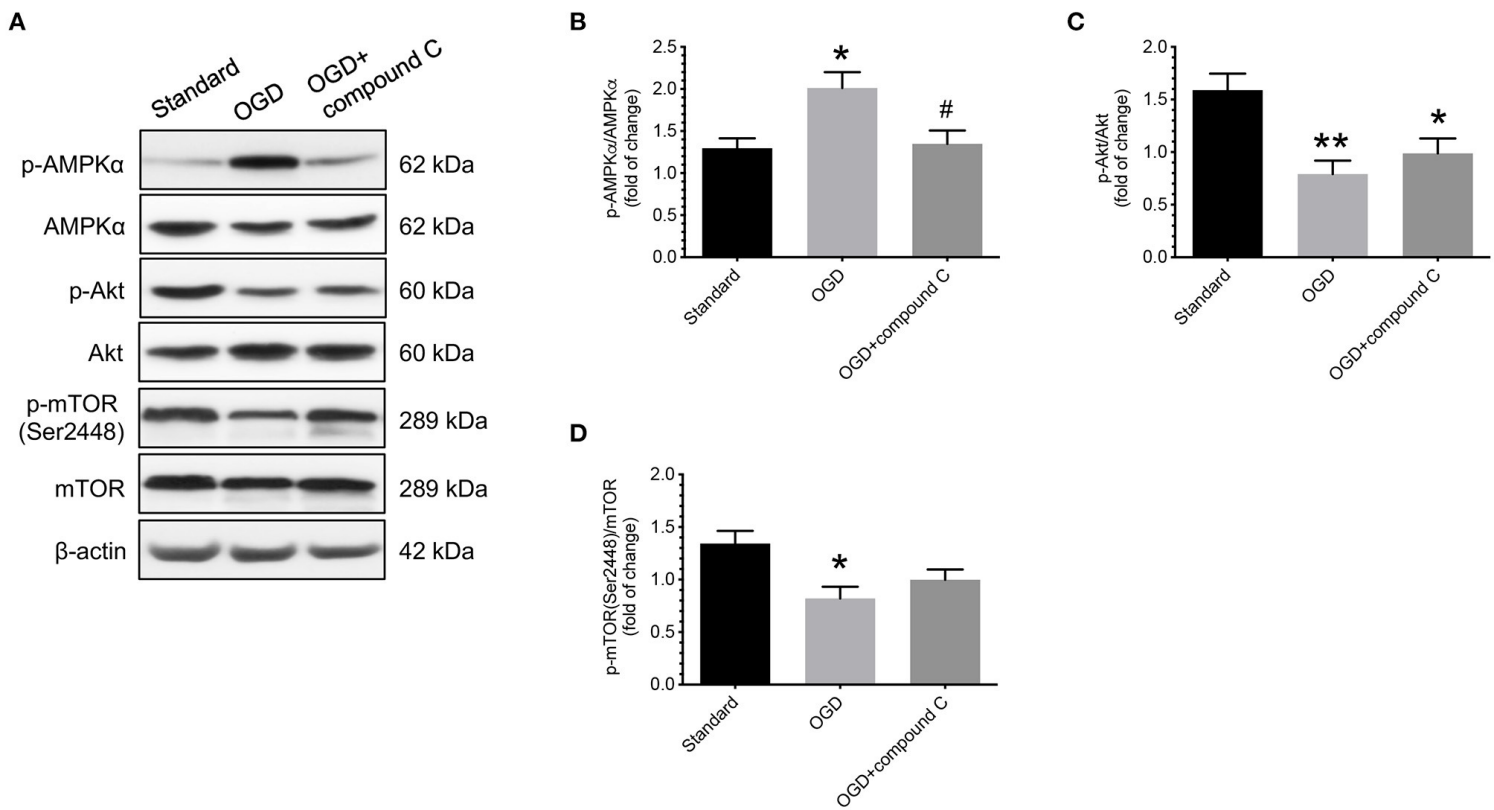
Effects of compound C on phosphorylation levels of Akt and mTOR in human cytotrophoblast cells under OGD conditions. Under OGD condition, administering compound C, a specific inhibitor of AMPK, did not cause a significant difference in the phosphorylation levels of Akt compared to the vehicle control. There was an increase in mTOR phosphorylation levels, though not statistically significant, when compound C was administered, as compared to the vehicle controls. **(A)** Representative blots show the levels of phosphorylated and total forms of AMPKα, Akt, and mTOR. β-actin was used to normalize loading variability. **(B–D)** Data are presented as mean ± SEM; *n* = 9 for each group. *, *P* < 0.05; **, *P* < 0.01, compared to cells cultured at standard conditions; ^#^, *P* < 0.05, compared with vehicle controls; based on one-way analysis of variance followed by Tukey's *post-hoc* test. Standard, standard culture conditions; OGD, vehicle controls under oxygen-glucose deprivation.

After demonstrating that there was an increase in the levels of AMPKα phosphorylation in cytotrophoblast cells treated with OGD conditions, we then studied the effects of administering compound C, a specific inhibitor of AMPK, on the changes in Akt and mTOR phosphorylation levels under OGD conditions. As shown in Figure 5, administering compound C did not cause a significant difference in the phosphorylation levels of Akt compared to the vehicle controls (Figure 5C). However, there was an increase in mTOR phosphorylation levels, though not statistically significant, when compound C was administered, as compared to the vehicle controls (Figure 5D).

### Effects of LY294002 on Phosphorylation Levels of AMPKα and mTOR in Cells Under Hyperglycemic Conditions

**Figure 6:**
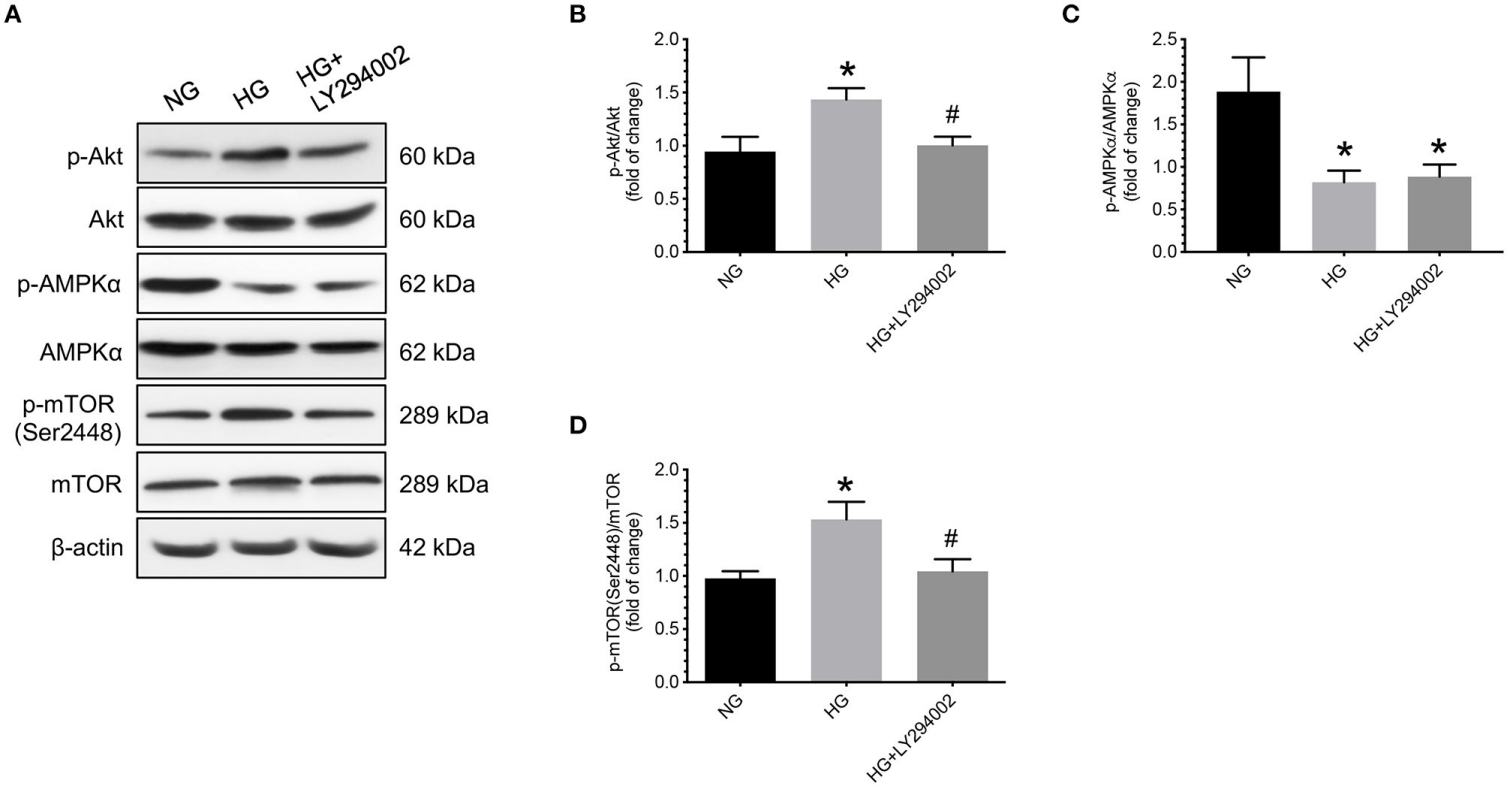
Effects of LY294002 on phosphorylation levels of AMPKα and mTOR in human cytotrophoblast cells under hyperglycemic conditions. Under hyperglycemic conditions, administering LY294002, a specific inhibitor of PI3K/Akt, caused a significant decrease in the levels of p-mTOR (Ser2448) compared to the vehicle control groups. Nevertheless, no significant differences in the levels of total and phosphorylated AMPKα were found between cells treated with or without LY294002. **(A)** Representative blots show the levels of phosphorylated and total forms of Akt, AMPKα, and mTOR. β-actin was used to normalize loading variability. **(B–D)** Data are presented as mean ± SEM; *n* = 9 for each group. *, *P* < 0.05, compared to cells cultured at normoglycemic conditions; ^#^, *P* < 0.05, compared to vehicle controls; based on one-way analysis of variance followed by Tukey's *post-hoc* test. NG, normoglycemic conditions; HG, vehicle controls under hyperglycemic conditions.

In contrast, under hyperglycemic conditions, administering LY294002, a specific inhibitor of PI3K/Akt, caused a significant decrease in the levels of p-Akt and p-mTOR (Ser2448) compared to the vehicle controls (Figures 6A,B,D). Nevertheless, no significant differences in the levels of total and phosphorylated AMPKα were found between cells treated with or without LY294002 (Figure 6C).

## Discussion

In this study, we found that (1) women with FGR pregnancies had lower levels of p-Akt, higher levels of p-AMPKα, and decreased phosphorylation of mTOR in their placentas than those in normal pregnant women. In contrast, GDM women with LGA infants had higher placental p-Akt, lower p-AMPKα, and higher p-mTOR levels than normal pregnant women; (2) primary cytotrophoblast cells subjected to OGD had lower p-Akt, higher p-AMPKα, and lower p-mTOR levels than cells cultured under standard conditions; (3) in contrast, increasing glucose concentrations led to higher levels of p-Akt and lower levels of p-AMPKα, and increased phosphorylation of mTOR than those observed under standard conditions. Together, these results suggest that Akt and AMPK signaling pathways are involved in regulating trophoblast mTORC1 activity in pregnancies complicated by FGR and GDM with LGA infants.

The most widely recognized predisposing factors for idiopathic FGR are defective extravillous cytotrophoblast invasion of the endometrium and incomplete transformation of maternal spiral arteries during the first trimester of pregnancy, which lead to impaired perfusion of the intervillous space and cause profound changes in prevailing tissue oxygen and glucose concentrations and nutrient supply (6). Several studies have reported a decrease in both levels of total and phosphorylated Akt or its activity in the placentas of women with FGR compared to that of normal pregnant women (20, 30–32). We did not find a significant difference in the placental levels of total Akt between pregnancies with and without FGR. However, we did notice less phosphorylation of Akt in the placentas from women with pregnancies complicated by FGR than that of normal pregnant women. This discrepancy is likely caused by differences in the definition and severity of FGR. In previous studies, FGR pregnancies were generally associated with abnormal umbilical artery flow patterns and a mean birth weight of approximately 30–50% of that in women in the control group (20, 30–32). On the other hand, most women with FGR pregnancies in our study had normal umbilical artery flow velocimetry and had a mean birth weight of approximately 70% of that in women with normal pregnancies. Nevertheless, our findings of placental changes in total and phosphorylated Akt levels are supported by observations from several animal studies, including dexamethasone-induced FGR in rats and maternal nutrition restriction-induced FGR in baboons (33–35).

Unlike Akt, only a few studies with inconsistent results compared the changes in AMPK and its phosphorylation in the placentas between women with normal pregnancies and those with pregnancies complicated by FGR (32, 36). Similar to a previous report from women living at high altitude with FGR (36), we found increased levels of phosphorylated AMPK in the placentas of women with FGR pregnancies than in those of women with normal pregnancies, although the same change was not observed in another report (32). Inconsistent results also exist between studies using different animal models of FGR (35, 37). Mice subjected to chronic hypoxia had higher AMPKα mRNA and p-AMPK levels in the placenta than those kept under normoxic conditions, whereas no differences were found in the placental levels of total and phosphorylated AMPK between pregnant baboons treated with and without nutritional restrictions (35, 37). Thus, the significance of AMPK activation in the placenta of FGR pregnancies remains unclear. Using human placental explants, Landau et al. demonstrated that pharmacological activation of AMPK induced mitochondrial toxicity and led to the impairment of cellular metabolism, including reduction of fatty acid uptake and glycolysis (38). In contrast, Lane et al. found that the activation of placental AMPK partially protected against hypoxia-induced FGR in mice (39). The protective role of AMPK was supported by knockdown experiments with interfering and hairpin RNAs in the mouse trophoblast stem cell line (40). Inhibition of AMPK activity was associated with the loss of appropriate placental differentiation, alterations in cell morphology, and reduction in cell growth and nutrient transport. Therefore, further studies are needed to clarify the significance of AMPK signaling in placental changes in pregnancies complicated by FGR.

The presence of differential changes in total and phosphorylated Akt levels in the placentas of women with GDM pregnancies compared to those with normal pregnancies remains controversial. Some studies showed that there was a decrease in total and phosphorylated Akt levels, or an increase in Akt mRNA levels, but no differences in the p-Akt levels (32, 41), while we and others found increased p-Akt levels but no change in the total Akt level in the placentas of GDM women compared to those of women without GDM (42–44). Indeed, in mouse models of GDM created by feeding a high-fat diet or genetic deficiency of leptin receptors, inhibition of placental Akt activity was associated with improved glucose tolerance and suppression of mRNA expression of pro-inflammatory cytokines and chemokines in the placentas (45, 46), suggesting that placental Akt plays a role in the pathophysiology of GDM pregnancy.

The reported changes in AMPK levels in the placentas of pregnancies complicated by GDM are also conflicting. In addition to our study, prior studies also found that women with GDM had lower placental levels of AMPK and p-AMPK than women with a normal pregnancy (22, 47–49), but others did not find similar changes and even found a reverse relationship (32, 50). A possible explanation for this discrepancy is due to the difference in the diagnostic criteria (one or two-step methods) or severity of GDM (nutritional therapy alone or combined with insulin therapy) across different studies. It has been demonstrated that, compared to women with a normal pregnancy, the placental levels of AMPK and p-AMPK were significantly lower in GDM women with AGA fetuses, and the differences became more profound in GDM women with macrosomia (47).

The dysregulation of placental mTOR plays an essential role in the pathophysiology of pregnancy complications, such as FGR and GDM. Similar to previous reports (12, 19–21, 23, 32), we found that there were differential changes in the placental levels of mTOR phosphorylation between women with normal pregnancies and those with pregnancies complicated by FGR or GDM with LGA infants. We further explored the mechanisms underlying the regulation of trophoblast mTOR expression using *in vitro* culture experiments. Using OGD as a condition mimicking the profound change in placental perfusion in pregnancies complicated by FGR, we found increased endoplasmic reticulum stress with decreased mTOR activity in cytotrophoblast cells (12). Here, we further demonstrated that cytotrophoblast cells subjected to OGD conditions had lower p-Akt, and higher p-AMPKα and p-TSC2 levels than the cells cultured under standard conditions. These changes were associated with decreased phosphorylation of mTOR. However, administering compound C, a specific AMPK inhibitor, did not cause significant changes in the phosphorylation of Akt, although partial reversal of mTOR phosphorylation was noted. These results suggest that, under these experimental conditions, AMPK is unlikely to be an upstream regulator of Akt, and AMPK and Akt are likely to independently exert their effects on mTOR. Alternatively, other factors, such as growth factors and reactive oxygen species, could play more crucial roles than AMPK in the regulation of Akt activity. mTOR plays an essential role in the control of protein synthesis, cell proliferation, and metabolism (6, 18). Decreased mTOR can increase apoptosis, autophagy of trophoblasts, and reduction of protein synthesis and nutrient transport in the placenta, leading to a smaller placenta and suboptimal growth of the fetus than that in women with normal pregnancies.

On the other hand, cytotrophoblast cells cultured under hyperglycemic conditions had higher p-Akt and lower p-AMPKα levels, which were associated with less p-TSC2 and more p-mTOR, compared with cells maintained under standard or hyperosmotic culture conditions. Furthermore, administering LY294002, a specific inhibitor of PI3K/Akt, decreased the levels of p-mTOR, but did not significantly change the levels of total and phosphorylated AMPKα compared to the vehicle controls. These findings indicate that Akt and AMPK likely have independent effects on mTOR phosphorylation under hyperglycemic conditions. Increased mTOR activity under hyperglycemic conditions can lead to increased protein synthesis, cell proliferation, and inhibition of apoptosis and autophagy, which subsequently lead to increased placental mass as seen in pregnancies complicated by GDM with LGA infants. Indeed, our prior study showed that GDM women with LGA infants had decreased placental apoptosis and autophagy compared to normal pregnant women with AGA infants (13).

In conclusion, we found differential changes in the placental levels of total and phosphorylated Akt, AMPKα, and mTOR among women with normal pregnancies and those with pregnancies complicated by FGR or GDM with LGA infants. By subjecting primary cytotrophoblast cells to OGD or hyperglycemic conditions, we further confirmed that Akt and AMPK signaling pathways are involved in regulating trophoblast mTOR activity in pregnancies complicated by FGR and GDM with LGA infants. These results shed lights on our understanding of the pathophysiology of these pregnancy complications and can form the basis of preventative strategies, although on the basis of hypothesis rather than scientific evidence of efficacy at the cellular level.

## Data Availability Statement

The raw data supporting the conclusions of this article will be made available by the authors, without undue reservation.

## Ethics Statement

The studies involving human participants were reviewed and approved by the Institutional Review Board of Chang Gung Memorial Hospital. The patients/participants provided their written informed consent to participate in this study.

## Author Contributions

T-HH, C-PW, and S-FC contributed to conception and design of the study. T-HH and S-FC performed the statistical analysis. T-HH wrote the first draft of the manuscript. All authors contributed to manuscript revision, read, and approved the submitted version.

## Funding

This research was funded by grants from the Ministry of Science and Technology, Taiwan (109-2314-B-182A-097-MY3 to T-HH and 108-2320-B-182-038-MY3 to C-PW) and Chang Gung Memorial Hospital (CMRPG1J0073 and CORPG1L0051 to T-HH and BMRPC17, CMRPD1L0051, and CMRPD1K0391 to C-PW).

## Conflict of Interest

The authors declare that the research was conducted in the absence of any commercial or financial relationships that could be construed as a potential conflict of interest.

## Publisher's Note

All claims expressed in this article are solely those of the authors and do not necessarily represent those of their affiliated organizations, or those of the publisher, the editors and the reviewers. Any product that may be evaluated in this article, or claim that may be made by its manufacturer, is not guaranteed or endorsed by the publisher.
