# Supplementary material for: Differential Changes in Akt and AMPK Phosphorylation Regulating mTOR Activity in the Placentas of Pregnancies Complicated by Fetal Growth Restriction and Gestational Diabetes Mellitus With Large-For-Gestational Age Infants
Source: Front Med (Lausanne). 2021 Dec 6;8:788969. doi: 10.3389/fmed.2021.788969 (PMC8685227; doi:10.3389/fmed.2021.788969)
Supplement: Supplementary file 1 [file Data_Sheet_1.docx]

**Supplementary Figure legends**

**Supplementary Figure 1. Effects of increasing glucose concentrations on the changes of 4E-BP1 and p70S6K phosphorylation in human cytotrophoblast cells.** Cytotrophoblast cells were isolated from normal term placentas and cultured under normoglycemic, hyperosmotic, and hyperglycemic conditions for 24 h, and the levels of phosphorylated and total forms of mTORC1 downstream proteins 4E-BP1 and p70S6K were compared. Increasing glucose concentrations led to higher levels of p-4E-BP1 (Thr37/46) and p-4E-BP1 (Thr70) and the ratios between phosphorylated and total form of 4E-BP1 (a to c). Hyperglycemia also caused a higher p-p70S6K (Thr389)/p70S6K than the normoglycemic and hyperosmotic conditions (a and d). (a) Representative blots show the levels of phosphorylated and total forms of 4E-BP1 and p70S6K. β-actin was used to normalize loading variability. (b, c, and d) Data are presented as mean ± SEM; n=9 for each group. *, *P*< 0.05, compared with cells cultured at normoglycemic conditions; ^#^, *P*< 0.05, compared with cells cultured at hyperosmotic conditions, based on one-way analysis of variance followed by Tukey’s *post-hoc* test. NG, normoglycemic conditions; OC, hyperosmotic conditions; and HG, hyperglycemic conditions.

**Supplementary Figure 2. Effects of OGD and increasing glucose concentrations on the changes of Raptor phosphorylation in human cytotrophoblast cells.** Primary cytotrophoblast cells were cultured under (a) standard or OGD conditions or (b) normoglycemic, hyperosmotic, and hyperglycemic conditions for 24 h, and the levels of phosphorylated and total forms of Raptor were compared. (a) OGD caused a higher p-Raptor (Ser792)/Raptor compared with the standard culture conditions. β-actin was used to normalize loading variability. Data are presented as mean ± SEM; n=8 for each group.*, *P*< 0.05; compared with cells cultured at standard conditions based on Student’s t-test. Standard, standard culture conditions; OGD, oxygen-glucose deprivation. (b) In contrast, increasing D-glucose concentrations led to lower levels of p-Raptor (Ser792) than that of those under normoglycemic and hyperosmotic conditions. β-actin was used to normalize loading variability. Data are presented as mean ± SEM; n=9 for each group. **, *P* < 0.01; compared with cells cultured at normoglycemic conditions; ^###^, *P*< 0.001, compared with cells cultured at hyperosmotic conditions. NG, normoglycemic conditions; OC, hyperosmotic conditions; and HG, hyperglycemic conditions.
