# Supplementary figures and images for: Differential Changes in Akt and AMPK Phosphorylation Regulating mTOR Activity in the Placentas of Pregnancies Complicated by Fetal Growth Restriction and Gestational Diabetes Mellitus With Large-For-Gestational Age Infants
Source: Front Med (Lausanne). 2021 Dec 6;8:788969. doi: 10.3389/fmed.2021.788969 (PMC8685227; doi:10.3389/fmed.2021.788969)

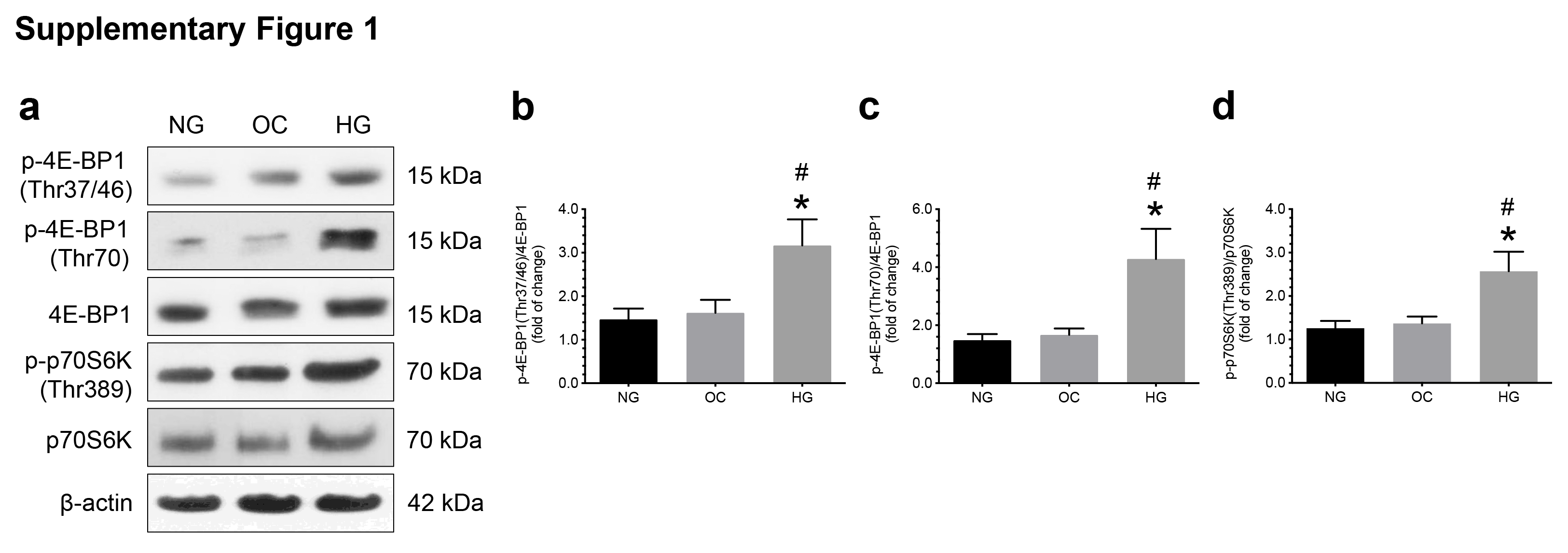

Supplement: Supplementary file 2 [file Image_1.TIF]

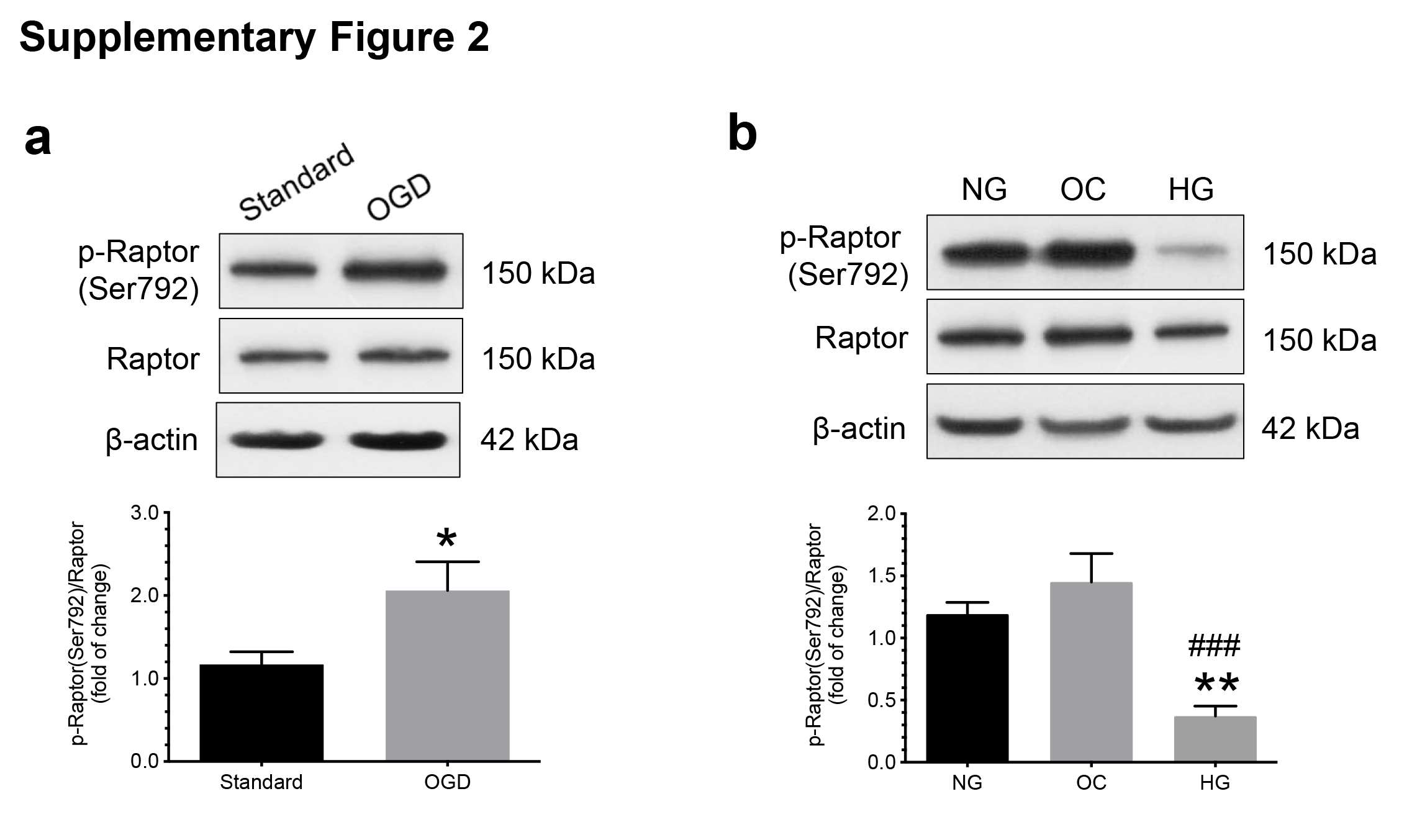

Supplement: Supplementary file 3 [file Image_2.TIF]
